# Spinal cord stimulation for chronic intractable trunk or limb pain: study protocol for a Chinese multicenter randomized withdrawal trial (CITRIP study)

**DOI:** 10.1186/s13063-020-04768-3

**Published:** 2020-10-07

**Authors:** Yang Lu, Peng Mao, Guihuai Wang, Wei Tao, Donglin Xiong, Ke Ma, Rongchun Li, Dan Feng, Wanru Duan, Shun Li, Zhijian Fu, Zhiying Feng, Yi Jin, Li Wan, Yan Lu, Daying Zhang, Bifa Fan, James Jin Wang, Luming Li

**Affiliations:** 1 Department of Neurosurgery, Beijing Tsinghua Changgung Hospital, School of Clinical Medicine, Tsinghua University, Beijing, China; 2 National Engineering Laboratory for Neuromodulation, School of Aerospace Engineering, Tsinghua University, Beijing, China; 3 Department of Pain Medicine, China-Japan Friendship Hospital, Beijing, China; 4 Department of Neurosurgery, Shenzhen University General Hospital, Shenzhen, China; 5 Department of Pain Medicine, Huazhong University of Science and Technology of Union Shenzhen Hospital, Shenzhen, China; 6 Department of Algology, Xinhua Hospital, Shanghai Jiaotong University School of Medicine, Shanghai, China; 7 Department of Pain Management, Wuhan Pu’ai Hospital, Tongji Medical College, Huazhong University of Science and Technology, Wuhan, China; 8 Department of Pain Management, Wuhan No. 1 Hospital, Wuhan, China; 9 Department of Neurosurgery, Xuanwu Hospital, Capital Medical University, Beijing, China; 10 Department of Pain, Zhejiang Provincial People’s Hospital, People’s Hospital of Hangzhou Medical College, Hangzhou, China; 11 Department of Pain Management, Shandong Provincial Hospital Affiliated to Shandong University, Jinan, China; 12 Department of Pain Medicine, The First Affiliated Hospital of Medical School of Zhejiang University, Hangzhou, China; 13 Department of Anesthesiology, Nanjing Jinling Hospital, Nanjing, China; 14 Department of Pain Management, The Second Affiliated Hospital of Guangzhou Medical University, Guangzhou, China; 15 Department of Pain Medicine, Xijing Hospital, Fourth Military Medical University, Xi’an, China; 16 Department of Pain Medicine, The First Affiliated Hospital of Nanchang University, Nanchang, China

**Keywords:** Chronic intractable pain, Spinal cord stimulation, Efficacy, Safety, Randomized controlled trial

## Abstract

**Background:**

Although effective results of many studies support the use of spinal cord stimulation in chronic pain patients, no randomized controlled trial has been undertaken in China to date. CITRIP is a multicenter, prospective, randomized, withdrawal study designed to evaluate the clinical effectiveness and safety of spinal cord stimulation plus remote programming management in patients with intractable trunk or limb pain.

**Method:**

Participants will be recruited in approximately 10 centers across China. Eligible participants with intractable trunk or limb and an average visual analog scale (VAS) score ≥ 5 will undergo a spinal cord stimulation test. Participants with VAS score reduction ≥ 50% could move forward to receive implantation of an implanted pulse generator. In the withdrawal period at 3-month follow-up visit, participants randomized to the experimental group (EG) will undergo continuous stimulation while ceasing the stimulation in the control group (CG). The outcome assessment will occur at baseline and at 1, 3 (pre- and post-randomization), and 6 months. The primary outcome is the difference of maximal VAS score between EG and CG in the withdrawal period compared with baseline before the withdrawal period. Additional outcomes include VAS score change at 1-, 3-, and 6-month follow-ups; responder rate (VAS score improving by 50%); achievement rate of a desirable pain state (VAS score ≤ 4); awake times during sleep; Beck Depression Inventory for depression evaluation; short-form 36 for quality of life evaluation; drug usage; and satisfaction rating of the device. Adverse events will be collected. The primary analysis will follow the intention-to-treat principle.

**Discussion:**

The CITRIP study seeks to evaluate the effectiveness and safety of a randomized withdrawal trial of spinal cord stimulation for patients with intractable trunk or limb pain.

**Trial registration:**

ClinicalTrials.gov NCT03858790. Registered on March 1, 2019, retrospectively registered

## Background

Chronic pain is a major clinical, social, and economic problem that appears to have a great negative influence on the quality of life. In 2010, chronic pain costs the USA approximately $635 billion in health care, which exceeds the total amount of cancer and cardiovascular and cerebral vascular diseases [1].

Chronic intractable pain (CIP) refers to the pain lasting for over 3 months and is refractory to conservative treatments, including oral medications, nerve block, epidural corticosteroids, physical and psychological rehabilitation therapy, and chiropractic care [2]. Since 1967, spinal cord stimulation (SCS) was used to manage chronic intractable pain, such as failed back surgery syndrome (FBSS) [3, 4], complex regional pain syndrome [5], and ischemic vascular disease-related pain [6]. Some systematic reviews also suggested that SCS has strong evidence for treating axial back/lumbar radiculopathy, neuralgia, and complex regional pain syndrome with long-term cost-effectiveness when compared with alternative treatment modalities [7, 8]. It is estimated that over 45,000 SCS procedures have been completed in the USA and over 80,000 worldwidely, with the demand on the uprising.

Several high-quality studies proved that around 50% of the patients could achieve long-term (at least 2 years) 50% pain relief in traditional low-frequency stimulation [9, 10]. Better results were shown in the newly developed stimulation settings such as 10-kHz high-frequency, in which 80% of the patients maintained at least 50% pain relief over 2 years [11]. Continued technological development of SCS (such as electrode specifications, electrode configurations, programming parameters) keeps extending the new indications that used to be refractory to the therapies into the competence circle of physicians [3, 4, 12]. While SCS continues to benefit an increasing number of pain patients in developed areas of the world, there is a small group of chronic pain sufferers that could access to the therapy in China.

There are only over 200 cases (excluding temporary lead implantation) being performed in China. Factors contributing to this situation include poor affordability of patients, ignorance of patients, and a tremendous knowledge gap with only few doctors in the core cities being able to implement this procedure. Thus, RCT evidence is needed to confirm the efficacy and safety of SCS in Chinese chronic intractable pain patients.

## Method/design

### Type of trial

The CITRIP study is a standard double-blind, placebo-controlled, enriched-enrollment, randomized-withdrawal (EERW) study to evaluate the clinical effectiveness and safety of SCS using G122R implanted pulse generator (IPG; PINS, Inc., Beijing, BJ, China) on chronic intractable trunk or limb pain patients. The EERW design was proposed for the analgesic drugs in chronic noncancer pain [13] and as a well-accepted design for the evaluation of chronic pain (systematic review [14]; several studies using EERW [15–18]). The main difference between EERW and classic RCT is to shift the point of randomization. The enrichment enrollment of the design shifts our focus to the individual “true” responders (defined as both achieving at least 50% pain intensity reduction and tolerating the treatment for 12 weeks) [14], which is important in the clinical practice.

**Fig. 1:**
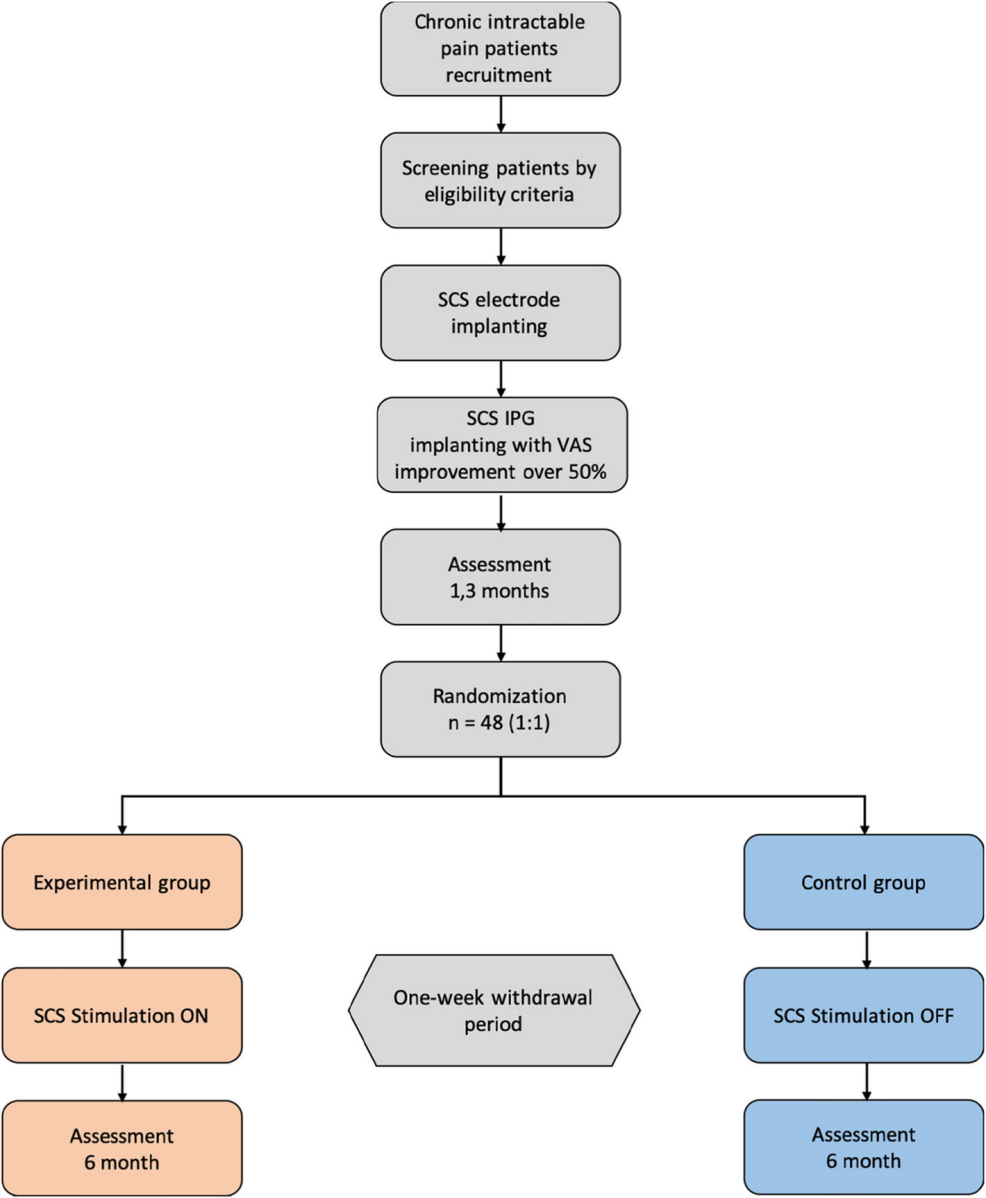
Flow chart of the CITRIP study

**Fig. 2:**
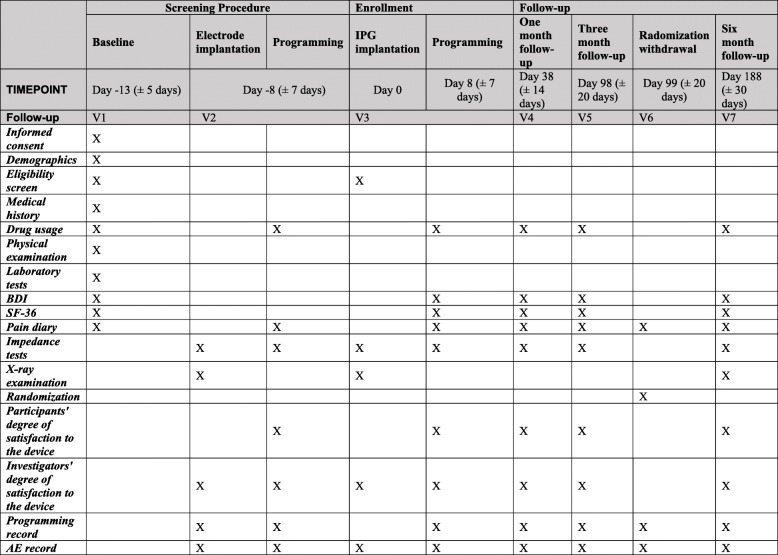
Standard Protocol Items (SPIRIT) for the CITRIP study

According to the trial flow chart (Fig. 1), the participants with chronic pain will be screened strictly based on the inclusion and exclusion criteria. Participants will receive the temporary spinal cord stimulation, and the eligible subjects could undertake the implantation of IPG. After a 3-month visit (V6), participants will be randomized (1:1) to one of the two groups: the experimental group (EG, stimulation continued in the withdrawal period) or the control group (CG, stimulation ceased in the withdrawal period). The clinical measurements of the two groups are shown based on Fig. 2. The participants in EG will continue to receive SCS therapy for 7 days, while the participants in CG will temporarily cease the SCS therapy during 1-week withdrawal period, or turn on the device in advance if the VAS score increases to the pre-operative baseline. All outcomes will be calculated by blinded statisticians appointed by the clinical research institute of Peking University following the end of the trial.

The CITRIP study protocol was written in accordance with the Standard Protocol Items: Recommendations for Interventional Trials Statement (SPIRIT). A SPIRIT checklist is included in Additional file 1. The trial will be carried out according to the principles of the Declaration of Helsinki (Edinburgh version, 2000).

### Study setting

The trial will be conducted at approximately 10 investigative centers domestically. Additional centers may be added during the course of the study. Every center should complete at least 4 cases of IPG implantation. No single center will exceed 30% of all enrolled patients. The protocol has been approved by the Clinical Trial Ethics Committee of China-Japan Friendship Hospital (protocol number 2019-135-Q17-2; date January 23, 2019) and registered on ClinicalTrials.gov protocol system (Clinical Trials Identifier: NCT03858790).

### Selection of patients

The study population comprises of patients suffering from chronic intractable trunk or limb pain. A patient must meet all of the inclusion criteria and none of the exclusion criteria to be eligible for the study. China has a large population of chronic intractable trunk or limb pain patients. The enrollment is predicted to come to a halt before December 2019.

### Inclusion criteria

Inclusion criteria are shown in Table 1.

**Table 1:** Inclusion criteria of the CITRIP study

Inclusion criteria	
1. Chronic intractable pain that lasts at least 3 months and refractory to the conservative therapies, including oral medications, nerve block, epidural corticosteroids, physical and psychological rehabilitation therapy, and chiropractic care	
2. Participant’s age is over 18	
3. Participants with good compliance and can complete post-operative follow-up	
4. Participants can understand the method and sign the informed consent	

### Exclusion criteria

Exclusion criteria are shown in Table 2.

**Table 2:** Exclusion criteria of the CITRIP study

Exclusion criteria	
1. Pregnancy, breast feeding, plan to be pregnant, or unwilling to use contraceptive methods	
2. Bleeding complications or coagulation disorders	
3. Severe mental or cognitive disorders, cannot cooperate during surgery and post-operative programming	
4. Life expectancy less than 1 year	
5. Need therapy or examination that could not be implanted with IPG (e.g., MRI, thermo-therapy)	
6. Pain reduction less than 50% during the trial period or cannot tolerate SCS	
7. Other inappropriate situations determined by investigators	

### Interventions

Pain treatment will be evaluated. To confirm the eligibility of the participants, the investigator should review the participant’s history of therapies for pain. Intractable pain means pain that is resistant to pharmacological agents or interventional therapies (for example, nerve block injections, radiofrequency, acupuncture, functional restoration, physical therapy, and psychological interventions, such as cognitive behavioral therapy) or intrathecal drug delivery or surgery.

Participants will undergo an SCS trial period (screening test, 4 days minimum, 15 days maximum). This screening test will be conducted with one or two percutaneous lead(s) or paddle leads. If successful (VAS score improves over 50% compared with baseline), an implanted pulse generator will be implanted. Participants who cannot accept the paresthesia or refuse to proceed the IPG implantation should also be considered as failing the screening test. After implantation, devices will be programmed to the optimal programming parameters and can be adjusted with the patient programmer, within the settings programmed in the clinic. Subjects will be provided with a patient programmer manual and will be instructed on the proper use and handling of the patient programmer.

#### Implant procedure

Consistent with the clinical practice, in the trial period, the electrode implantation was undertaken in the awake surgery fashion. The subject was brought to a conscious sedated state while maintaining local anesthesia. An external pulse generator (T802, PINS, Inc., Beijing, BJ, China) was utilized to stimulate the spinal cord using the percutaneous or paddle lead with the guidance of fluoroscopy. The subject should provide verbal feedback regarding paresthesia coverage of painful regions. Lead was always repositioned to achieve better paresthesia overlap of painful areas. Once 50% reduction of pain was attained during the trial period, an IPG implantation surgery was subsequently performed. The leads, anchored to the supraspinous ligaments, were tunneled to the pocket site and connected to the IPG. Intraoperative impedance testing ensured electrical integrity. A subcutaneous pocket was created in the buttock, chest, or abdomen according to the physician and subject’s preference.

### Lead programming

After lead implantation, investigators will review the contact combination in a consecutive manner. The paresthesia coverage and related lead configuration, amplitude, pulse width, and frequency will be recorded. The optimal contact combination (usually the best coverage with the lowest output) will be used. After IPG implantation, investigators are recommended to program 4 sets of lead configurations (2 paresthesia sets and 2 paresthesia-free sets) for the participants according to the programming record in the screening phase. The programming will be completed before discharging the hospital. Participants are allowed to change among the predetermined configurations by themselves (switch the presetting configurations or increase/decrease the amplitude within a presetting limit). The following must be underlined during the programming:

1. Programming sessions should not exceed 1 h in case the fatigue leads to the cooperation problem or subjective bias.
2. Paresthesia configuration is firstly recommended for the investigators; paresthesia-free configuration usually requires more frequent recharges.

### Remote programming

In China, there are only 200–300 cases of SCS being implanted every year. Only a few doctors in central cities of different areas are trained to implement this surgical technique. China is a large country with 1.3 billion people, most of whom lived in the underdeveloped areas. Based on the remote programming system developed for deep brain stimulation [19, 20], the remote programming function has been well equipped for the SCS system and will be used in this trial to facilitate the post-operative management of participants.

For a short introduction, the remote system could enable the doctors to implement the programming of the SCS system at any time in patients with network coverage. IPG will connect with the patient’s cell phone by the Bluetooth at the same time doctor’s computer connecting with the patient’s cell phone. Real-time video interaction will give doctors sufficient information to adjust the configuration and parameters of the SCS.

In this trial, participants could have access to the doctors via this remote programming system at home or at a local clinic. It provides participants convenience with less expenditure. As for the use of the remote system, several issues should be stressed:

1. All participants and at least one caregiver are ensured to be familiar with the remote programming and be able to cooperate with investigators to complete the whole procedure before leaving the hospital.
2. Participants could apply for the remote programming within V4-V7. Investigators will adjust the configuration and parameters of the SCS based on the initial programming records.
3. If the therapeutic effect is not satisfactory as in the hospital, an on-site programming is recommended.

### Randomization and withdrawal

Randomization allocation will be concealed from the investigators and participants and implemented with a central automatic web-based data management system of the third party (CIMS, Ltd., Chengdu, China). Permuted blocks and stratification will be used to generate the randomization assignments, in order to keep the balance of patients in each center. The stratification is based on the medical center to which the participant belongs.

After a 3-month visit, subjects will run into the 7-day withdrawal phase. They will be randomly assigned to the experimental group (EG, turning on the SCS) or control group (CG, turning off the SCS) in a 1:1 ratio, according to the “random number table” generated by the CIMS clinical trial central randomization system of the third party. For the nature of spinal cord stimulation, the trial could not be strictly double-blinded (SCS therapy induces paresthesia). In order to carry out the trial under unbiased conditions with investigators and participants as much as possible, the participant will not be told to which group he/she is assigned. Also, each center designates a specific investigator to access to the randomization system with his own password and account. When a certain subject needs to run into the randomization phase, this investigator enters the subject’s trial number and gets the unique random number of this subject from the randomization center, which assigns the subject to EG or CG. This designated investigator who could access to the randomization system is the only unblinded investigator during the blind phase. He/she is responsible for the turning on/off of the device, modulation of the parameters, and checking whether the VAS score returns to the baseline. Other investigators, particularly the evaluators, will be blind to the treatment conditions until the end of the open-label period. The blinded investigators will be trained to obey the experimental rules and not enquire into the randomized results.

Participants randomized to the EG continued the SCS stimulation-on, while participants in the CG will turn off the SCS during the 7-day withdrawal period. CG participants will turn on the SCS if the VAS score returns to the pre-operative baseline and lasting for 2 days. Otherwise, turning on the SCS is on the 8th day post-randomization.

### Primary outcome

The primary outcome will be the difference of maximal visual analog scale (VAS) score between EG and CG in the withdrawal period compared with the baseline in the withdrawal period. The VAS is a subjective evaluation scale for pain rating. Participants will record their trunk and limb pain using a (paper) pain diary every day for a 7-day withdrawal period. For the withdrawal baseline and other follow-up visits, participants will record a 4-day period of VAS score. The proportion of subjects in each group with 50% reduction in average VAS score at the trial period (V2) compared with pre-operative baseline will undertake the IPG implantation and will be calculated.

The primary outcome calculation formula is:

1 $$ \left({\mathrm{VAS}}_{\mathrm{EGbase}}-{\mathrm{VAS}}_{\mathrm{EGwd}}\right)-\left({\mathrm{VAS}}_{\mathrm{CGbase}}-{\mathrm{VAS}}_{\mathrm{CGwd}}\right) $$

where VAS_EGbase_ is the baseline 4-day average VAS score of EG before the withdrawal period, VAS_EGwd_ is the maximal VAS score of EG in the withdrawal period, VAS_CGbase_ is the baseline 4-day average VAS score of CG before the withdrawal period, and VAS_CGwd_ is the maximal VAS score of CG in the withdrawal period.

### Secondary outcomes and safety outcomes

The secondary outcomes include VAS score change at 1-, 3-, and 6-month follow-ups; responder rate (VAS score improving by 50%); achievement rate of a desirable pain state (no worse than mild pain, VAS scores ≤ 4) [21]; awake times during sleep; Beck Depression Inventory for depression evaluation [22]; short-form 36 for quality of life evaluation [23]; drug usage; and satisfaction rating of the device (a modified Patients’ Global Impression of Change scale [24], which consists of PGIC items and ratings for the satisfaction for the device).

The occurrence rate of AE/SAE during the different periods of the trial will be used to evaluate the safety of SCS. The investigators are responsible for the management of participants’ AEs and SAEs. The clinician will turn off the SCS therapy and evaluate the vital signs of the participant if the SAE occurs, then the related therapy would be given for the participant. The investigators should determine whether the IPG and/or electrode should be removed or replaced according to the event. All the above events must be reported to the trial manager within 24 h.

### Sample size and power calculations

The primary comparison is the difference between EG and CG in terms of VAS score in the withdrawal period. According to the results of the study [25], the clinically important outcome of VAS score is 20 mm at most, which does not vary with gender, age, cause, or severity of pain [26, 27]. A sample size of 48 (24 per arm) achieves 80% power to detect the significant difference using a two-sided, two-sample *t* test at a significance level (alpha) of 0.025. We assumed the true difference between the means to be 0 and a standard deviation of the outcome of 2.6. Thus, the sample size calculation formula was:

2 $$ n=\frac{2{\left({Z}_{1-\alpha /2}+{Z}_{1-\beta}\right)}^2{\delta}^2}{{\left(\left|D\right|-\Delta  \right)}^2} $$

where |*D*| is the expected mean difference of the two groups, *∆* is the superiority threshold value (0, here), *Z* is the quantile of the standard normal distribution, *α* is the first class error level for the statistical test (0.025, here for unilateral test; 0.05 for bilateral test if need), and *β* is the second class error level for the statistical test (0.2, here). The calculated sample size per group through this calculation is 21. Considering that the maximum possible loss rate is 10%, the total sample size is 24 pairs (*N* = 48) [24].

These 48 cases will use the G122R rechargeable IPG combined with percutaneous leads (PINS, Inc., Beijing, BJ, China). For the non-rechargeable model G122 and paddle lead, other 6 participants will be recruited for each condition (3 cases for each). No statistical requirements are needed for these 6 cases. In summary, the final sample size is 54 (24 pairs plus extra 6).

To implement within-group comparisons, data will be analyzed with *t* tests and *χ*^2^ tests for continuous variables and categorical variables, respectively. The Kolmogorov-Smirnov test or parametric tests will be performed with the data of normal distributions. Rank-sum test will be used for the non-parametric data. The factors that affect curative effects will be analyzed with a multinomial logistic regression method. A *p* value < 0.05 will be considered as statistically significant. A validated statistical software package will be used for the analyses of the study results (for example, SAS).

### Quality control and trial monitoring

Clinical documents have been formulated before the implementation of the trial with the discussion of the clinical experts from different medical centers, including standard operating procedure (SOP), investigator’s brochure, and detailed research plan. All staff participating in this program have received the meticulously organized training including different parts of patient enrollment, surgery technique, programming demonstration, database demonstration, and CRF completion. All the investigators from every center should complete the consistency training held by China-Japan Friendship Hospital.

As for the assessment, because of the nature of spinal cord stimulation, the trial could not be strictly blinded (SCS therapy induces paresthesia). In order to minimize the bias, participants will fill the pain diary without study staff consultation or visibility. Also, the resulted VAS score is an average score of the consecutive 4-day ratings (except the VAS score in the withdrawal period, which is the maximal score).

To quantify performance bias, the drug treatment and addition/removal of SCS of all the participants are followed during the whole study. The drug usage from the baseline to the end of the study will be compared in order to interpret the between-group outcome differences.

Data is firstly monitored by the investigator himself/herself to ensure the authenticity and completeness. Then the certificated clinical research associate (CRA) will monitor, audit, and ensure the trial is conducted and data are generated, documented (recorded), and transported in compliance with the protocol, GCP, and regulatory requirements. CRA will do the monitoring and quality control of the study on behalf of the sponsors (PINS Medical, Ltd., Beijing, China). Monthly checks will be implemented on the CRF of each participant, data accuracy, protocol compliance, and violation issues. The quality control facility of each center will ensure the study’s quality with the processes of the program usually for 3 times at the first enrollment, half the enrollment, and end of the enrollment.

An independent clinical committee consisting of a minimum of three independent clinicians will review all the adverse events and discuss and determine any relationship to the SCS therapy.

The final report will follow the Consolidated Standards of Reporting Trials (CONSORT) extension guidelines for non-pharmaceutical interventions.

## Discussion

The past five decades have witnessed the development of SCS from a conception to a well-established therapy, named as “the last resort therapy” for chronic intractable pain. Numerous studies have demonstrated that SCS is clinically effective for the chronic intractable pain induced by failed back surgery syndrome, complex regional pain syndrome, periphery ischemic pain, refractory angina pectoris, and many other diseases [3–6, 13]. Here, we propose this first SCS trial in China using a randomized withdrawal design demonstrating that the SCS is well tolerated and effective for the management of chronic intractable pain. The outcome measures selected for this trial are based on the previous RCTs of SCS [10] and IMMPACT recommendations [28, 29].

Double-blind, placebo-controlled, enriched-enrollment, randomized-withdrawal (EERW) design usually has the screening phase, titration phase, and maintenance of effective dose and randomization withdrawal phase [14]. Just like other pain drugs focused on the central or peripheral mechanism of pain, SCS is regarded as the “electroceuticals” to reduce pain with several mechanisms [14, 30, 31]. The programmable and reversible characteristics make it perfectly fit into the EERW study design. In clinical practice, pain doctors usually perform a screening procedure for the patients to test whether the IPG should be implanted (“natural” screening phase, in which participants who get 50% or more reduction of the pain are candidates for the IPG implantation) [32]. Patients will receive a thorough programming to achieve the best pain relieve (titration phase). In order to get the “true responders,” we set the maintenance phase as 3 months to stabilize the therapeutic effect of SCS before randomization. Three months after implantation is usually enough for the parameter settlement and a stable therapeutic effect (maintenance of effective dose). Since almost all the intractable chronic pain patients’ symptoms will return within 24 h after turning off the SCS [33], we choose to have the 7 days of withdrawal period to avoid a carry-over effect in this study. Besides, the randomized withdrawal period may also have contributed to the study sensitivity, as it allowed patients who achieved good pain control to experience a loss of pain control. Sensitivity and awareness of loss of pain control could be a more profound experience than the gain in pain control that occurs with conventional studies. Thus, with the idea of electroceutical and design of EERW, this trial could provide more insights relevant to clinical practice.

In 2018, there have been less than 200 SCS cases (IPG implantation) completed in China. There is a big knowledge gap for the doctors and patients. To implement the SCS trial, a standard training program will be proceeded with the help of China-Japan Friendship Hospital, whose PI is well trained in USA and also a pioneer in this field in China. The sponsor has to make sure that all the investigators are capable of carrying out the trial protocol, especially performing the surgery and post-operative programming.

Besides lack of the expertise, China is a big country with the area of 9,600,000 km^2^ and people of 1.39 billion. The Gross Domestic Product (GDP) per capita in 2018 is $9376.97, one sixth of US’s. For this development level, post-operative programming is still a big burden for the neuromodulation patients. As for the neuromodulation therapies, surgery is only a beginning, with post-operative management being crucial. In particular, the uneven distribution of medical resources makes it even harder for the patients in the remote areas of China. To solve the challenges above, telemedicine could be used to overcome these healthcare barriers including travel distance, shortage in expertise distribution, growing disability or immobility, and lack of accessibility [34, 35].

Based on the remote programming mode we developed for our deep brain stimulation system [19, 20], SCS has been equipped with the remote programming mode. With the well-developed 4G network infrastructure in China, participants could connect directly to the expertise to receive the pain evaluation and programming. Chronic pain is well suited to telemedicine because it could be visually and vocally assessed. Furthermore, to stabilize the therapeutic effect of SCS, telemedicine is associated with higher patient satisfaction [36] and quality indicators [37].

It is important to note that this study has several limitations. Firstly, all the outcome measurements are patient-reported outcomes from a pain diary. The study could be biased with the placebo effect. Another potential limitation of this trial is the follow-up duration of 6 months. We noted that a longer follow-up for assessing chronic SCS therapy over 1 year remains valuable and helpful to reduce the possible placebo effect.

## Trial status

Enrollment of participants into this study started in February 2019 and continues. Target enrollment for this study is 54 participants.

## Protocol version

Version 1.2 (November 29, 2018)

## Supplementary information

**Additional file 1:.** SPIRIT 2013 Checklist: Recommended items to address in this clinical trial protocol and related documents. It is strongly recommended that this checklist is read in conjunction with the SPIRIT 2013 Explanation & Elaboration for important clarification on the items. Amendments to the protocol should be tracked and dated. The SPIRIT checklist is copyrighted by the SPIRIT Group under the Creative Commons “Attribution-NonCommercialNoDerivs 3.0 Unported” license.

## Data Availability

Data from this study are unavailable before the end of this trial. Data collected during the course of the research will be kept strictly confidential and only accessed by members of the trial team. All participants will be allocated an identification number, and participant’s details will be stored on a secure database of a third party (CIMS, Ltd., Chengdu, China). The datasets analyzed during the current study are available from the sponsor on reasonable request.

## Abbreviations

SCS: Spinal cord stimulation
IPG: Implantable pulse generator
CRF: Case report form
EG: Experimental group
CG: Control group
AE: Adverse event
SAE: Serious adverse event
SOP: Standard operating procedure
CRA: Clinical research associate
